# Occurrence data of terrestrial vertebrates of Son Tra Peninsula, Da Nang City, Vietnam

**DOI:** 10.3897/BDJ.7.e39233

**Published:** 2019-11-27

**Authors:** Bang Van Tran, Duy Le, Huy Quoc Hoang, Duc Minh Hoang

**Affiliations:** 1 Southern Institute of Ecology, Vietnam Academy of Science and Technology, Ho Chi Minh, Vietnam Southern Institute of Ecology, Vietnam Academy of Science and Technology Ho Chi Minh Vietnam; 2 GreenViet Biodiversity Conservation Center, Da Nang, Vietnam GreenViet Biodiversity Conservation Center Da Nang Vietnam

**Keywords:** distribution, occurrence, mammal, aves, reptile, amphibian

## Abstract

**Background:**

The forest of Son Tra Peninsula was designated as a nature reserve in 1977 and serves as a green lung for Da Nang City. Due to the economic development scheme of Da Nang City, the forest of the peninsula has been disturbed by human activities and by invasive plant species. Moreover, the management board of the nature reserve lacked sufficient data of the species distribution of its biodiversity for developing future management and conservation plans. To provide and enhance knowledge for the distribution of wildlife species in Son Tra Peninsula, we conducted field surveys over two years to collect data on species richness and distribution and then build a biodiversity database for the protected area.

**New information:**

The project collected the occurrence data of 145 species of terrestrial vertebrates, accounting for 51.6% of vertebrate species known from the peninsula with total of 900 observations. In addition, distribution data of six threatened species were recorded on the peninsula.

## Introduction

Son Tra Peninsula of Da Nang City is well known as a home for one of the most beautiful primates, the red-shanked douc (*Pygathrix nemaeus*) and the forest is considered as the “green lung” of the city. Located on the peninsula, Son Tra Nature Reserve was established in 1977 to protect the forest and its biodiversity in an area of ca. 4,000 ha. The nature reserve then was reduced to 3,871 ha in the “National Planning for Biodiversity Conservation toward 2020 and vision to 2030”. In addition, the peninsula was planned for national tourism development with an area of 1,056 ha from 2016. Hence, the total area allocated for conserving the habitat for wildlife was reduced to 3,383 ha. Therefore, the forestry area, as well as the biodiversity of the peninsula, including the nature reserve, will be impacted under the pressure of economic development for the city.

Before the re-unification of Vietnam, the fauna of Son Tra was surveyed and 23 mammal species were reported for the peninsula (Jones 1983, van Peenen et al. 1971). Although the nature reserve was established in 1977, its wildlife was not inventoried until 1997 when the occurrence of 174 terrestrial vertebrate species, including 36 mammal species, 106 bird species, 23 reptile species and 9 amphibian species, was reported (Dinh et al. 1997). Recently, a study on the herpetofauna of the nature reserve reported the presence of 18 amphibian and 52 reptile species (Phan et al. 2014). Several opportunistic observations and studies conducted at the nature reserve have contributed to the knowledge of species richness of the fauna of the nature reserve (Lippold and Vu 2008, Dinh et al. 2010, Streicher and Ulibarri 2014). After revising the terrestrial fauna list by removing synonyms and unconfirmed species, the terrestrial vertebrates of Son Tra consists of 41 mammals, 151 birds, 52 reptiles and 18 amphibians. Although the previous studies provided initial information to understand the biodiversity of the nature reserve, data on the distribution of animals including coordinates were lacking or not sufficient for planning conservation activities, particularly in light of the high pressure caused by human activities. In addition, after almost 20 years, the biodiversity of the Son Tra Peninsula has likely changed due to the invasive species *Merremia* spp. Therefore, this study provides crucial data on species distribution of confirmed species that occur within the nature reserve.

## Project description

### Title

Research on conservation and restoration of terrestrial and marine ecosystems at Son Tra Nature Reserve, Da Nang City. Project code: 26/15 – ĐTĐL.CN - XHTN

### Personnel

Project manager: Dr Duc Minh Hoang

Project coordinatior: Mr. Bang Van Tran

Other principle members of the project are: Dr Truong Hong Luu, Dr. Long Ngoc Vu, Mr. Bach Xuan Le Nguyen, Mr. Hoa Xuan Nguyen, Dr. Vy Xuan Nguyen, Asso. Prof. Minh Van Vo, Dr. Long Thang Ha and Mr. Thien Quang Huynh.

### Study area description

The project was carried out on Son Tra Peninsula focusing on Son Tra Nature Reserve, Da Nang City. In addition, the project also covers the marine ecosystem surrounding the Son Tra Nature Reserve.

### Design description

The project was designed in three steps. Firstly, the status of land cover and distribution of important marine ecosystems (seagrass and coral reef) was mapped by using current satellite images. After that, a database of the biodiversity of the nature reserve, including terrestrial and marine ecosystems based on field surveys data, was transferred to local authorities. Lastly, based on the results of the project, one sustainable management plan will be recommended to the management board of the nature reserve, as well as for the peninsula.

### Funding

Project was funded by the Ministry of Science and Technology, Vietnam. Funding Number 26/15-DTĐ.CN-XH.

## Sampling methods

### Study extent

The project was conducted at Son Tra Peninsula where Son Tra Nature Reserve is located. The forest of the peninsula, based on satellite images from 2016, comprises seven habitat types: poor evergreen broadleaf forest (1,872 ha), medium evergreen broadleaf forest (1,445 ha), planted forest (252 ha), bare land/grassland with *Merremia* spp. (226 ha); bare land/other grassland (368 ha); agricultural land/other (118 ha) and residential land (807 ha). Mammals, birds, reptiles and amphibians are our targeted taxa. The fieldwork was conducted from 2016 to 2017 with 46 survey-days.

### Sampling description

The mammal fauna was studied by applying three methods, including visual survey in both day and night-time to record easily recognised species, mist nets were used for capturing bats and box traps were used for capturing small mammals like rodents and shrews. While the visual surveys were conducted along the roads and available trails in the forest, mist nets and box traps were set up near streams. The visual survey was also the main method to collect bird data by using binoculars and a camera with telephoto lens for taking the photos of species. The herpetofauna was surveyed in both day and night-time to maximise results. Surveyors focused on streams together with the roads to observe and capture animals by hand. The surveys usually took place from 06h00 till noon, from 14h00 to 17h00 and from 19h00 to 23h00.

### Quality control

Controlling data: Each observation of an animal contained fundamental information such as location (coordinates and altitude), habitat and name of surveyor. The coordinates and altitude of locality were derived from hand-held Garmin GPS (model 64 CSX). The habitat type was based on the current classification system for land cover of Son Tra Peninsula as provided in the section of study extent. Regarding species identification, herpetofauna species and small mammals, such as bats and rodents, were identified based on examination of specimens. A Guide to the Mammals of South-East Asia (Francis 2008) was used to identify easily recognisable species, such as members of Carnivora, Primates and Ungulates. To identify bats, we used the reference “Bats of Vietnam: Checklist and an identification manual” (Kruskop 2013) and recent determination keys on the bats of Vietnam. For rodent identification, “An Identification Guide to the Rodent of Vietnam” (Lunde and Nguyen 2005) was used. The main reference for identifying bird species was “A Field Guide to the Birds of South-East Asia” (Robson 2014). To identify amphibian and reptile species, “Herpetofauna of Vietnam” (Nguyen et al. 2009) was used along with the latest references on herpetofauna studies in Vietnam.

Controlling nomenclature name of species: To ensure using the updated scientific name and common name of species, we double-checked the nomenclature of species with online databases: Mammal species of the world (Wilson and Reeder 2005) (https://www.departments.bucknell.edu/biology/resources/msw3), Bird databases (https://www.hbw.com/species), Reptile databases (http://reptile-database.reptarium.cz) (Uetz and Hosek 2019) and Amphibian Species of the world (http://research.amnh.org/vz/herpetology/amphibia) (Frost 2019). In addition, the species were also checked in the database of IUCN Red List of Threatened species (https://www.iucnredlist.org) for scientific names and conservation status of species.

## Geographic coverage

### Description

**Figure 1.**
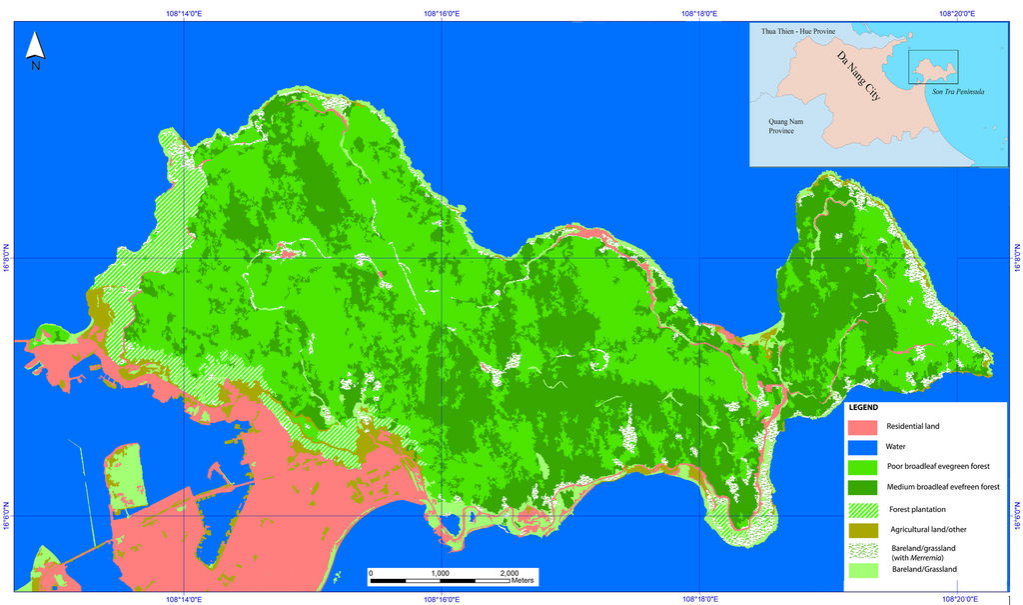
The land cover map of Son Tra Nature Reserve

Son Tra Nature Reserve is located in Son Tra Peninsula, which is situated on the north-east edge of Da Nang City. The geographic coordinates of the peninsula are 10°06' N to 10°09' N and 108°13' E to 108°21' E (Fig. 1). The topography of the nature reserve consists of mountainous areas with north-south slopes with north slopes being steeper than those of the south. The nature reserve is strongly divided by a system of small streams. The peak of Son Tra Nature Reserve has a height of 696 m a.s.l. and the other two peaks are at an elevation of 647 m and 621 m. The land area below 200 m a.s.l. accounts for 57.96% of the nature reserve, while the land above 400 m covers 12.46%. There is a road system that allows access to both low and high areas of the nature reserve.

### Coordinates

 and Latitude; and Longitude.

## Taxonomic coverage

### Description

The occurrence data contained the distribution location for 145 species, belonging to four classes: Mammalia (20 species), Aves (82 species), Reptilia (27 species) and Amphibia (16 species). There is a total of 900 records, including 128 records for the mammals, 445 records for birds, 145 records for reptiles and 182 records for amphibians (Suppl. material 1). The data include occurrences of six species that are listed as Near Threatened, Vulnerable and Endangered in IUCN Red List of Threatened species (Table 1).

## Temporal coverage

### Notes

The project was conducted from January 2015 to December 2018. Data were collected within the project timeline during four field trips in April 2016, October 2016, May 2017 and August 2017.

## Usage rights

### Use license

Creative Commons Public Domain Waiver (CC-Zero)

## Data resources

### Data package title

Occurrence data of terrestrial vertebrates of Son Tra Peninsula, Danang City, Vietnam

### Resource link

https://data.depositar.io/en/dataset/occurence-data

### Number of data sets

1

### Data set 1.

#### Data set name

Occurrence data of terrestrial vertebrates of Son Tra Nature Reserve, Vietnam

#### Data format

csv

#### Number of columns

34

#### Download URL

https://data.depositar.io/en/dataset/e1c93cbd-9af4-4a5f-bea7-0e264ace5dbf/resource/aee7c690-1f5c-4997-a872-1a8b79eec12f/download/occurrence_SonTra_Peninsula_final.csv

#### Description

The data were prepared by following DARWIN CORE.

**Data set 1.** 

Column label	Column description
occurrenceID	The Globally Unique Identifier number for the recored
basisOfRecord	The specific nature of the data record: PreservedSpecimen, HumanObservation
eventDate	date format as YYYY-MM-DD
year	Year of the event was recorded
month	The month of the event was recorded
date	The date of the event was recorded
verbatimEventDate	The verbatim original representation of the date and time information for an Event: the format of date as MM/DD/YYYY
scientificName	The full scientific name including the genus name and the lowest level of taxonomic rank with the authority
kingdom	The full scientific name of the kingdom in which the taxon is classified
phylum	The full scientific name of the phylum or division in which the taxon is classified.
class	The full scientific name of the class in which the taxon is classified
order	The full scientific name of the order in which the taxon is classified
family	The full scientific name of the family in which the taxon is classified
genus	The full scientific name of the genus in which the taxon is classified
specificEpithet	The name of the first or species epithet of the scientificName
scientificNameAuthorship	The authorship information with the date for the scientificName formatted
taxonRank	The taxonomic rank of the most specific name in the scientificName
identifiedBy	Name of identifier to the taxon that recorded
dateIdentified	The date on which the subject was identified as representing the Taxon.
decimalLatitude	The verbatim original latitude of the Location
decimalLongitude	The verbatim original longitude of the Location
geodeticDatum	The geodetic datum for coordinates: WGS84
verbatimCoordinateSystem	The spatial coordinate system for the verbatimLatitude and verbatimLongitude: decimal degrees
continent	The name of the continent in which the Location occurs
country	The name of the country or major administrative unit in which the Location occurs
countryCode	The standard code for the country in which the Location occurs: VN
stateProvince	The name of the next smaller administrative region than country: Danang
county	The full, unabbreviated name of the next smaller administrative region than stateProvince: Son Tra
locality	Name of the Nature Reserve: Son Tra Nature Reserve
language	A language of the resource: English
institutionCode	The acronym of name of Southern Institute of Ecology, SIE
collectionCode	The name, acronym, coden or initialism identifying the collection or dataset from which the record was derived: SIEZC
individualCount	the number of individuals that were recorded for the event
preparations	name of methods for preserving specimen

## Supplementary Material

69E476E9-98DB-5E7B-8DBE-24F03B1F306A 10.3897/BDJ.7.e39233.suppl1 Supplementary material 1 Occurrence data of terrestrial vertebrates in Son Tra Peninsula, Da Nang City, Vietnam Data type: Occurrence
Brief description: The file contains occurrence data of terrestrial vertebrate species in Son Tra Peninsula, Da Nang City. Data were organised in 14 columns and 901 rows with first row as the header.
File: oo_326518.xlsx https://binary.pensoft.net/file/326518 Bang Van Tran, Duc Minh Hoang, Huy Quoc Hoang, Duy Le

## Figures and Tables

**Table 1.** The records of globally-threatened species from Son Tra Peninsula.

Scientific name	Common name	IUCN Category	Number of records
*Ophiophagus hannah*	King cobra	VU	1
*Cuora mouhotii*	Keeled box turtle	EN	1
*Pitta nympha*	Fairy pitta	VU	3
*Psittacula alexandri*	Red-breasted parakeet	NT	1
*Pygathrix nemaeus*	Red-shanked douc	EN	35
*Nycticebus pygmaeus*	Pygmy slow loris	VU	2
